# Longitudinal Monitoring of Simulated Interstitial Fluid Pressure for Pancreatic Ductal Adenocarcinoma Patients Treated with Stereotactic Body Radiotherapy

**DOI:** 10.3390/cancers13174319

**Published:** 2021-08-26

**Authors:** Ramesh Paudyal, Eve LoCastro, Marsha Reyngold, Richard Kinh Do, Amaresha Shridhar Konar, Jung Hun Oh, Abhay Dave, Kenneth Yu, Karyn A. Goodman, Amita Shukla-Dave

**Affiliations:** 1 Department of Medical Physics, Memorial Sloan Kettering Cancer Center, New York, NY 10065, USA; paudyalr@mskcc.org (R.P.); locastre@mskcc.org (E.L.); konarsha@mskcc.org (A.S.K.); OhJ@mskcc.org (J.H.O.); 2 Department of Radiation Oncology, Memorial Sloan Kettering Cancer Center, New York, NY 10065, USA; ReyngolM@mskcc.org; 3 Department of Radiology, Memorial Sloan Kettering Cancer Center, New York, NY 10065, USA; dok@mskcc.org; 4 Touro College of Osteopathic Medicine, New York, NY 10027, USA; asdavea@gmail.com; 5 Department of Gastrointestinal Oncology Service, Memorial Sloan Kettering Cancer Center, New York, NY 10065, USA; YuK1@mskcc.org; 6 Tisch Cancer Institute at Mount Sinai Hospital, New York, NY 10029, USA; Karyn.Goodman@mountsinai.org

**Keywords:** computational fluid modeling, Darcy velocity, dynamic contrast-enhanced MRI, hydraulic conductivity, interstitial fluid pressure and velocity, Starling principle, Stereotactic body radiation therapy

## Abstract

**Simple Summary:**

High vessel permeability, poor perfusion, low lymphatic drainage, and dense abundant stroma elevate interstitial fluid pressures (IFP) in pancreatic ductal adenocarcinoma (PDAC). The present study aims to monitor longitudinal changes in simulated tumor IFP and velocity (IFV) values using a dynamic contrast-enhanced (DCE)-MRI-based computational fluid modeling (CFM) approach in PDAC. Nine PDAC patients underwent DCE-MRI acquisition on a 3-Tesla MRI scanner at pre-treatment (TX (0)), immediately after the first fraction of stereotactic body radiotherapy (SBRT, (D1-TX)), and six weeks post-TX (D2-TX). The partial differential equation of IFP formulated from the continuity equation using the Starling Principle of fluid exchange and Darcy velocity–pressure relationship was solved in COMSOL Multiphysics software to generate IFP and IFV parametric maps using relevant tumor tissue physiological parameters. Initial results suggest that after validation, IFP and IFV can be imaging biomarkers of early response to therapy that may guide precision medicine in PDAC.

**Abstract:**

The present study aims to monitor longitudinal changes in simulated tumor interstitial fluid pressure (IFP) and velocity (IFV) values using dynamic contrast-enhanced (DCE)-MRI-based computational fluid modeling (CFM) in pancreatic ductal adenocarcinoma (PDAC) patients. Nine PDAC patients underwent MRI, including DCE-MRI, on a 3-Tesla MRI scanner at pre-treatment (TX (0)), after the first fraction of stereotactic body radiotherapy (SBRT, (D1-TX)), and six weeks post-TX (D2-TX). The partial differential equation of IFP formulated from the continuity equation, incorporating the Starling Principle of fluid exchange, Darcy velocity, and volume transfer constant (K^trans^), was solved in COMSOL Multiphysics software to generate IFP and IFV maps. Tumor volume (V_t_), K^trans^, IFP, and IFV values were compared (Wilcoxon and Spearman) between the time- points. D2-TX K^trans^ values were significantly different from pre-TX and D1-TX (*p* < 0.05). The D1-TX and pre-TX mean IFV values exhibited a borderline significant difference (*p* = 0.08). The IFP values varying <3.0% between the three time-points were not significantly different (*p* > 0.05). V_t_ and IFP values were strongly positively correlated at pre-TX (ρ = 0.90, *p* = 0.005), while IFV exhibited a strong negative correlation at D1-TX (ρ = −0.74, *p* = 0.045). V_t_, K^trans^, IFP, and IFV hold promise as imaging biomarkers of early response to therapy in PDAC.

## 1. Introduction

Pancreatic ductal adenocarcinoma (PDAC) is currently the fourth highest cause of cancer death worldwide, with a 5-year survival rate of <7% [1]. PDAC is characterized by a dense collagenous stroma consisting of abundant extracellular matrix (ECM) and stromal cells, including cancer-associated fibroblasts (CAF), inflammatory cells, and endothelial cells [2]. Typically, more than 80% of PDAC tumor volume is composed of stromal cells and ECM. Related to ECM composition and deficient vasculature, PDAC manifests an increased interstitial fluid pressure (IFP) and decreased oxygenation, which can impact response to therapy [3]. The dense ECM facilitates cancer cell migration, tumor growth, and metastatic invasion and induces interstitial hypertension [4]. During growth and progression, tumors generate mechanical forces, which couple with aberrant tumor vessels, inducing abnormal solid and fluid stresses that hinder response to various treatments [5]. Solid stress can directly compress cancer, and stromal cells, whereas the blood and lymphatic vessels are indirectly compressed, inhibiting and driving tumor growth towards high- and low-stress regions, respectively [6,7]. The hydrostatic and osmotic pressure, which are due to the architecture of tumor vessels, shows an active process in the tumor stroma that also regulates IFP through the contractile characteristics of the ECM [8].

A prior study suggested that diagnosing most pancreatic cancers is effective before tumor size reaches 20 mm [9]. For most newly diagnosed PDAC patients, who present with localized, but unresectable disease, radiation therapy (RT) represents the only locoregional treatment option. Recent advances in stereotactic treatment planning technology and solutions for tracking internal organ motion have allowed for the delivery of ablative doses of RT to PDAC patients who meet the criteria for favorable outcomes [10,11,12]. However, response to RT varies significantly among individual patients and may be in part related to the unique characteristics of the PDAC tumor microenvironment, including hypoxia [2]. There is a need to develop and translate new imaging biomarkers that may predict early treatment efficacy to allow for better risk stratification and possible treatment intensification in appropriate candidates [13]. RT using high doses per fraction may be associated with rapid, immediate changes to the tumor microenvironment, including tumor cellularity and vascularity, which correlate with eventual clinical RT response [14,15]. Quantification of change to tumor vasculature using noninvasive biomarkers derived from dynamic contrast-enhanced magnetic resonance imaging (DCE-MRI) would enhance understanding of RT resistance mechanisms and improve patient risk stratification, impacting overall patient survival [16,17,18].

T_1_ weighted (T_1_w) DCE-MRI uses gadolinium (Gd)-based contrast agent (CA) to capture the change in target tissue signal intensity, which allows measuring essential characteristics of tumor vasculature [19]. Vessel leakiness influences the extent of CA uptake in tumor tissues where leaking blood vessels irrigate solid tumors. A previous clinical study reported that the pre-treatment (TX) volume transfer constant (K^trans^), a measure of perfusion and permeability, derived from the standard Tofts model and the change of K^trans^ (ΔK^trans^) after initiation of chemotherapy, was able to predict response to antiangiogenic therapy [20] and classify responding and nonresponding tumors in PDAC [21]. Recently, Do et al. utilized the fast-exchange regime (FXR) model to assess the ΔK^trans^ in PDAC patients treated with stereotactic body (SB)RT [22]. This study reported that the ΔK^trans^_1-0_ values were significantly different from those of ΔK^trans^_2-0_ values. In a preclinical study, Cao et al. reported that FXR derived K^trans^ could detect early responses to the stroma-directed drug, PEGPH20, in the animal tumors model of PDAC [23].

CA extravasates into interstitial space through the capillary walls and is regulated by the balance of hydrostatic and oncotic pressure described by the Starling principle of fluid exchange [24]. The structurally aberrant and permeable tumor blood vessels and lack of functioning lymphatic vessels elevate the IFP and approach microvascular pressure levels but cannot persistently elevate to the point of vessel compression [25]. Increased IFP is associated with increasing capillary hydraulic conductivity. Solid stress, capable of overcoming microvascular pressures, is mediated by ECM. Provenzano et al. identified that elevated intratumoral pressure in PDAC is associated with increased local production of hyaluronic acids [26]. Decrease in IFP from the central regions to the tumor boundary leads to exudate tissue fluid movement in porous media [27]. Interstitial fluid velocity (IFV) (i.e., called Darcy velocity) is the gradient of the local IFP and is very high where IFP drops precipitously at the tumor/normal tissue boundary. Increased IFP has been identified as one of the major obstacles to the uptake and distribution of therapeutic agents in solid tumors [6]. Therefore, IFP is the characterizing hallmark of solid tumors’ physical microenvironment that aids in developing targeted delivery therapies [28].

Different techniques for measuring tumor IFP have been developed and tested [29,30,31,32]. The gold standard procedure for IFP measurement is the invasive wick-in-needle (WIN) method [25,26,33]. Studies in head and neck and breast carcinomas’ tumor size exhibited significant positive correlations with IFP [34,35]. Clinical data showed evidence that tumor IFP correlates with response to treatment [36,37]. Previous studies have reported that pre-TX high tumor IFP and hypoxia in patients with locally advanced cervical cancer are independently associated with inferior survival after RT alone [36,38]. However, the use of the WIN method in critical tumor regions is constrained by direct accessibility.

Noninvasive estimation of tumor IFP would be a significant step in understanding the highly abnormal microenvironment of PDAC and its influence from the stroma and dense ECM [39,40]. Previous studies reported different approaches for evaluating tumor IFP using DCE-MRI in preclinical and clinical settings [16,39,41]. To assess the robustness of the approach, IFP computed from the DCE-MRI approach was compared with the standard WIN method [39,41]. The results of previous studies suggested that the microvascular and interstitial hydraulic conductivities influence the outcome of the porous media fluid flow simulation [41,42,43,44,45,46]. However, the correlations need further validation to be part of clinical practice.

The DCE-MRI-based computational fluid modeling (CFM) solves the 2nd order partial differential (PD) equation formulated for the biological tissue based on the mass conservation principle, consisting of sources and sinks. The 2nd order PD equation incorporates the Starling equation [24] and Darcy velocity–pressure relationship [47]. The CFM simulation utilizes the tumor tissue physiological parameters measured in pre-and clinical settings such as capillary and tissue hydraulic conductivities, including K^trans^ values as input parameters for simulation of IFP and IFV [45,46,48,49].To the best of our knowledge, the DCE-based CFM approach for estimating quantitative IFP and IFV values in patients with PDAC is still unexplored. Herein, DCE data acquired at three-time points (i.e., pre-treatment (TX), immediately after the first fraction of SBRT, and post-TX) in the previous study were used [22]. The present feasibility study aims to monitor longitudinal changes in simulated tumor IFP and IFV values at three-time points using the DCE-MRI-based CFM approach in patients with PDAC.

## 2. Materials and Methods

### 2.1. Patients

Our institutional review board approved this prospective longitudinal study, compliant with the Health Insurance Portability and Accountability Act (HIPAA). Written informed consent was obtained from all eligible patients diagnosed with PDAC. Criteria for study inclusion required patients 18 years of age or older having a diagnosis of PDAC to be treated with stereotactic body radiation therapy (SBRT). The accrual period for this study was February 2016 to August 2018. The present study investigates a new data analysis method: DCE-MRI-based CFM estimates of IFP and IFV. Three-time points of DCE data were acquired at pre-treatment (TX(0)), immediately after the first fraction of stereotactic body radiotherapy (D1-TX(1)), and 6 weeks post-TX (Post-TX(2)) in nine patients (N = 9, M/F = 6/3, median age = 64 years, range = 55–67) with PDAC as detailed in Do et al. [22]. Three patients (N = 3) had a tumor in the head of the pancreas, one patient had a tumor in the head and body (N = 1), and five patients (N = 5) had a tumor in the body of the pancreas. As a note, one patient at pre-TX and another patient at D1-TX did not participate in the MRI study.

### 2.2. DCE Data Acquisition

T_1_w images were acquired using the fast 3D spoiled gradient recalled echo (SPGR) sequence for both pre-contrast T_1_ mapping (i.e., T_10_) and dynamic Gd-enhanced series (Gadobutrol (Gadavist, Bayer Health Care)). Twenty phase T_1_w images were acquired with repetition time/echo time = 5.6/2.3 milliseconds, flip angle (FA) = 15°, acquisition matrix = 231 × 116, FOV = 30–35 cm, and NS = 10–12. The temporal resolution was about <15.0 (s). DCE-MRI data were acquired with a series of multiple breath-holds as detailed by Do et al. [22].

### 2.3. DCE Data Analysis

**Figure 1:**
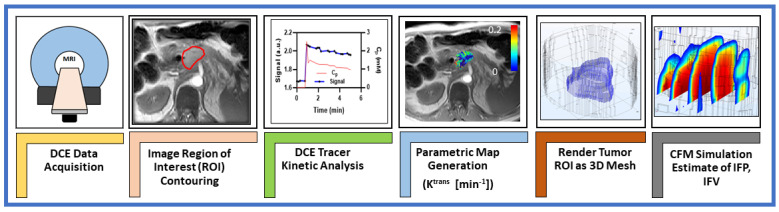
Representative workflow for MRI–based computational fluid modeling simulations.

The schematic workflow of DCE-MRI-based CFM analysis is illustrated in Figure 1.

The extended Tofts model (ETM) is used for modeling the tissue CA concentration with time data given by Equation (1) [50].

(1) $$C_{t}\left(t\right)=K^{\mathrm{trans}}\int_{0}^{t}e^{-k_{\mathrm{ep}}\left(t-\tau \right)}C_{p}(\tau )\;d\tau +{\;v}_{p}C_{p}\left(t\right),$$

where K^trans^ is the volume transfer constant; C_p_(t) is the CA concentration with time in vascular (plasma) space (i.e., arterial input function (AIF)), and v_p_ is the volume fraction of the vascular space. k_ep_ is defined as the transport rate constant of CA from the extravascular extracellular space (EES) to vascular space, expressed as k_ep_ = K^trans^/v_e_, where v_e_ is the EES volume fraction.

To determine the flux out of the solid tumor vasculature, the CA concentration profile in tissue was modeled using Equation (2), which accounts for diffusive and convective cases [42,51].

(2) $$v_{e}\frac{{\mathrm{dC}}_{e}}{\mathrm{dt}}=\frac{\mathrm{PS}}{V}\left(C_{p}-C_{e}\right)+\frac{J_{v}}{V}\left(1-\sigma_{T}\right),$$

where the (PS/V) and (J_v_/v) are related to diffusive and convective flux, respectively; P is the diffusive vascular permeability (m^2^); S/V is the capillary surface-to-volume ratio; C_e_ is CA concentration of the EES; J_v_/V is the filtration rate per unit volume, and σ_T_ is the osmotic reflection coefficient. Equation (2) provides estimates of three parameters: v_e_, PS/V, and J_v_/V.

The J_v_/V values estimated in Equation (2) can be rescaled to model spatially varying capillary hydraulic conductivity, $L_{p}$, with the literature value given by Equation (3) [43,52].

(3) $$L_{p}\left(\frac{S}{V}\right)=L_{p,0}\times \left(\frac{S}{V}\right)\times \frac{\frac{J_{v}}{V}}{<\frac{J_{v}}{V}>},$$

where 〈J_v_/V〉 represents the average of (J_v_/V). The literature values of capillary hydraulic conductivity, (L_p,0_) = 2.1 × 10^−11^ m/Pa.s and capillary surface-to-volume ratio (S/V) = 2.0 ×10 ^4^ m^−1^ were used [53].

### 2.4. Regions of Interest (ROIs)

Regions of Interest (ROIs) for K^trans^ estimation were manually delineated on the tumor lesion present in all slices in the late phases of T_1_w DCE images using ITK-SNAP [54], showing a clear tumor-to-normal tissue boundary. The tumor ROI volume (V_t_) was calculated from the T_1_w dynamic images.

The ROI was rendered as a 3D stereolithography (STL) mesh for later inclusion in the CFM simulation. Equations (1) and (2) were fitted on a voxel-by-voxel basis using an individual AIF extracted from the abdominal aorta. DCE image postprocessing and K^trans^ generation were performed using in-house developed software MRI-QAMPER [55].

### 2.5. Computational Fluid Modeling Theory and Analysis

The computational fluid modeling approach is based on the principles of fluid mechanics as described by the Navier–Stokes (NS) equations [56]. For an incompressible fluid (∇u = 0), NS reduces to a function that describes the fluid flow in porous media, relating the gradient IFP (∇p_i_) to IFV, which is referred to as the Darcy velocity, u’, and is given by Equation (4):

(4) $$u=-K_{H}\nabla p_{i},$$

Darcy velocity ‘u’ is a bulk fluid movement within the porous media. K_H_ is the local tissue hydraulic conductivity, assumed constant in this work. It is defined as the permeability ratio of the porous media, K (m^2^), over the interstitial fluid viscosity, μ (Pa˖s).

The net rate of CA flux across the capillary wall φ_v_, referred to as source term, is described by Starling’s principle relating the tumor hydrostatic and oncotic pressures and is given by Equation (5). Similarly, the flux from the lymphatic vessel, φ_L_, representing the sink term, can be expressed as follows (Equation (6)):

(5) $$\phi_{v}=L_{P}\frac{S}{V}\left(p_{V}-p_{i}-\sigma_{T}\left[\pi_{V}-\pi_{i}\right]\right),$$

(6) $$\phi_{L}=\{\begin{array}{c} \frac{L_{\mathrm{pL}}S_{L}}{V}\left(p_{i}-p_{L}\right)\\ 0,\;\mathrm{in}\;\mathrm{tumor} \end{array},\;\mathrm{in}\;\mathrm{normal}\;\mathrm{tissue}$$

where p_v_ and p_i_ are pressures in vascular and interstitial space, respectively; π_v_ and π_i_ are the tumor osmotic pressure of the plasma and the interstitial fluid; L_p_ is the hydraulic conductivity of the vessel wall; S/V is the surface to volume ratio of the capillary wall, and σ_T_ is the osmotic reflection coefficient. φ_L_ is the rate of net interstitial fluid removal by lymphatic drainage. However, φ_L_ is assumed to be negligible (≈0) in tumor tissue due to dysfunctional lymphatic vessel development [57,58]. The value for lymphatic pressure, $p_{L}$, taken from the literature, was set as a constant, near-zero value in normal tissue because $\phi_{L}$ fluid flux leaving tissue is effectively zero in the tumor domain.

The continuity equation, which enforces conservation of mass, consisting of the source and sink term in porous media, is given by Equation (7):

(7) $$\nabla \cdot u=\varphi_{v}-\varphi_{L}\to -K_{H}\nabla^{2}p_{i}=\varphi_{v}-\varphi_{L,}$$

Microscopically, the tumor vasculature is highly heterogeneous. CA enters passively into the EES via diffusion from the blood plasma in a hyperpermeable capillary, which is described by K^trans^. To account for the bulk flow of spatially varying CA into the EES within the tumor [43,44,45,49], the normalized K^trans^ value is incorporated into the continuity equation (Equation (8)), including the Starling equation of net flux change between vascular and lymphatic vessels. Note, a voxel-wise K^trans^ value was normalized and included in the equation as a factor to convey relative vascular leakiness.

The final observable continuity equation is simulated in terms of the dependent variable, interstitial pressure, p_i_, as a 2nd-order partial differential equation (PDE) given by Equation (8):

(8) $$-{\;K}_{H}\nabla^{2}p_{i}=\frac{K^{\mathrm{trans}}}{\langle K^{\mathrm{trans}}\rangle }[L_{P}\frac{S}{V}\left(p_{V}-p_{i}-\sigma_{T}\left(\pi_{V}-\pi_{i}\right)\right)]-\frac{L_{\mathrm{pL}}S_{L}}{V}\left(p_{i}-p_{L}\right),$$

The CFM simulation for Equation (8) was carried out across a patient-specific 3D mesh model of the tumor, generated from the ROIs used for DCE perfusion image analysis. The contoured tumor ROIs were converted to binary masks, and the tumor region was dilated by 10 pixels to incorporate normal surrounding healthy tissue into the model. The ROIs for tumors and normal surrounding tissue were resliced to 1 mm^3^-isotropic in MATLAB using NIfTI Toolbox [45,59] and converted to the STL file format. STL files were imported into the simulation software and interpreted as boundary meshes for the solution space of the model, incorporating a tumor with surrounding normal tissue to capture tissue boundary fluid dynamics. In the present study, a simplified geometry was used to represent a complex PDAC tumor structure.

K^trans^ maps were resliced to a matching isotropic space to ensure accurate spatial alignment with ROI meshing and interpolation, as detailed elsewhere [45,59]; K^trans^ and L_p_ × S/V values were imported as scalar fields on the mesh domain in COMSOL. Numerical values for physical constants were assigned to normal and tumor tissue to effectively describe the differences in tissue properties of the appropriate regions of the 3D STL domain mesh, as given below. These physiological values are taken from those previously cited in the literature [44,46,60]. To generate the IFP map, a stationary-state solution of the continuity PDE in Equation (8) was solved on the 3D extended domain ROI (including tumor and normal surrounding regions), with a no-flux condition at the outermost simulation boundary.

Herein, the simulation was conducted using the general coefficient form of the PDE module in a commercial multiphysics software package (COMSOL Inc., Stockholm, Sweden), which employs a finite element method to solve PDE equations for computations of IFP and IFV. The model parameters used for simulation are as follows: base vessel permeability L_p,0_ = 2 × 10^−11^ mPa^−1^s^−1^ (in tumor, t) and 3 × 10^−12^ mPa^−1^s^−1^ (in normal tissue, n) [44]; base microvascular surface area per unit volume (S/V) = 2 × 10^4^ (t), 7 × 10^3^ (n) m^−1^ [44]; lymphatic filtration coefficient (L_pL_S_L_/V) =1 × 10^−7^ Pa^−1^ s^−1^; hydraulic conductivity (K_H_) = 1.9 × 10^−12^ (t), 3.8 × 10^−13^ (n) m^2^ Pa^−1^ s^−1^; microvascular pressure (p_V_) = 2300 Pa; pressure of the lymphatic system ($p_{L}$) = 0 Pa; osmotic pressure in microvasculature (π_v_) = 2670 Pa [44]; osmotic pressure in interstitial space (π_i_) = 3230 (t), 1330 (n) Pa [60], and average osmotic reflection coefficient for plasma (σ_T_) = 0.82 (t), 0.91 (n) (Unitless) [42].

Quantitative IFP and IFV values were extracted from the simulation using the COMSOL MATLAB LiveLink interface to create the volume maps at each voxel coordinate of the dilated tumor ROI. Mean and standard deviation (SD) values were calculated in MATLAB for IFP and IFV over each voxel in tumor and normal tissue regions. The resulting maps were saved to NIfTI format and could be visually reviewed using ITK-SNAP [54].

### 2.6. Statistical Analysis

The data were reported as mean ± SD. Wilcoxon rank-sum test was used to compare the tumor volume (V_t_), IFP, and IFV between pre-TX, D1-TX, and D2-TX. To quantify the relationships between V_t_ with IFP and IFV, the Spearman correlation coefficient ρ, and the associated *p* values were calculated. The weak, moderate, and strong correlation coefficients were, respectively, categorized as ρ = < 0.3, ρ = 0.4–0.7, and ρ = 0.8–1.0 [61]. *p* < 0.05 was considered statistically significant in all cases. All statistical calculations were performed in the R computing environment [62].

## 3. Results

Tumor ROI mean volumes (V_t_) at pre-TX (23.22 ± 27.15 cm^3^ (N = 8)), D1-TX (16.86 ± 28.87 cm^3^ (N = 8)), and D2-TX (28.87 ± 30.60 cm^3^ (N = 9)) were not significantly different (*p* > 0.05). At pre-TX, V_t_ ranged from 1.08 to 68.53 cm^3^. Here, it is worthy to note that one patient at pre-TX and another patient at D1-TX had no MRI exams.

**Figure 2:**
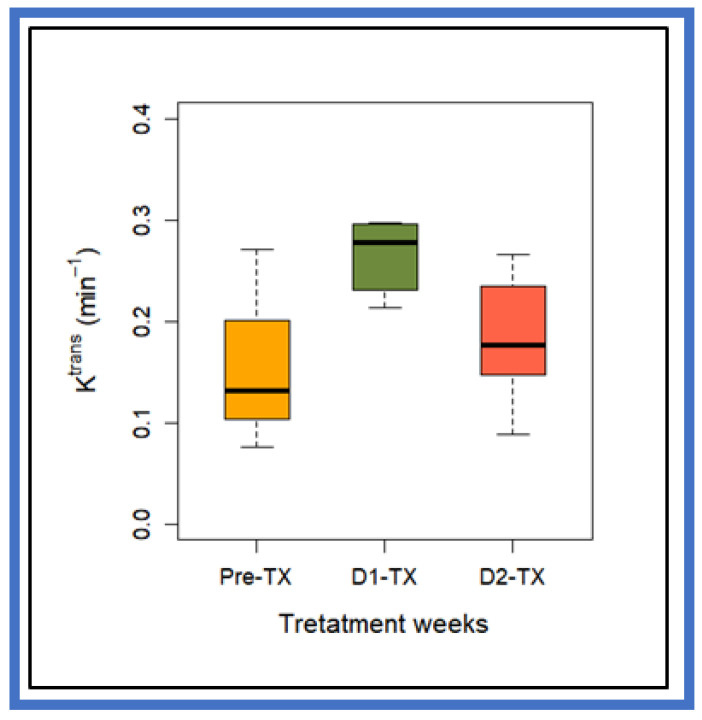
Box-and-whisker plot comparing volume transfer constant, K^trans^, values at pre-TX, D1-TX, and D2-TX in PDAC patients treated with stereotactic body radiation therapy. The horizontal black lines in boxes represent the median, and the top and bottom of boxes represent 25% and 75% percentiles of data values, respectively. Mean K^trans^ value at D1-TX was significantly different from pre-TX and D2-TX (*p* = 0.005 for Pre-TX and *p* = 0.01 for D2-TX). Pre-TX and post-TX K^trans^ values did not reach statistical significance (*p* > 0.05).

ETM-derived mean K^trans^ values between pre-TX and D1-TX (*p* = 0.005), and between D1-TX and D2-TX were significantly different (*p* = 0.01), while pre-TX and D2-TX mean values were not significantly different (*p* = 0.27) (Table 1). The box–whisker plot displays the K^trans^ values at pre-TX, D1-TX, and D2-TX (Figure 2).

**Figure 3:**
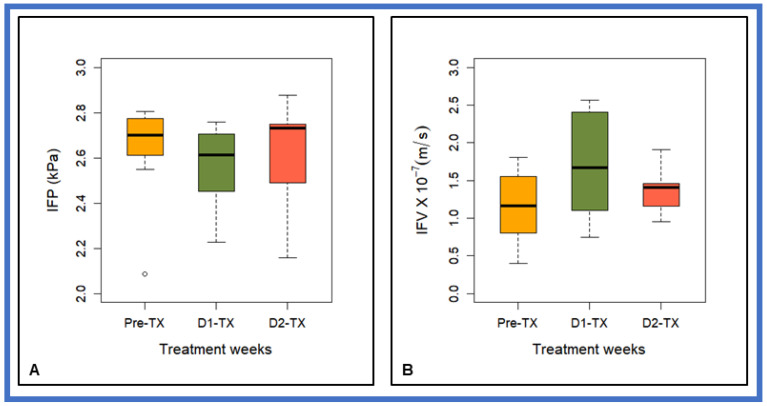
Box-and-whisker plot summarizing CFM estimates of (**A**) interstitial fluid pressure (IFP) and (**B**) interstitial fluid velocity (IFV) at pre-TX, D1-TX, and D2-TX in patients with PDAC treated with stereotactic body radiation therapy. The horizontal black lines in boxes represent the median, top, and bottom of boxes represent 25% and 75% percentiles of data values, respectively, and the open circle represents an outlier at pre-TX. Mean IFV values between the D1-TX and pre-TX showed a borderline significant difference (*p* = 0.08).

Figure 3 box- and- whisker plot displays the DCE-based CFM-estimated mean IFP and IFV values at three time-points (i.e., pre-TX, D1-TX, and D2-TX) from PDAC patients treated with SBRT. No statistically significant difference in mean IFP values was found between the three-time points (*p* > 0.05, Table 1). Mean IFV values between D2-TX and pre-TX, and D2-TX and D1-TX were not significantly different (*p* > 0.05). In contrast, D1-TX and Pre-TX mean IFV values showed a borderline significant difference (*p* = 0.08, Table 1). Still, there is considerable overlap between the IFP and IFV values at all three-time points.

The mean percentage (%) decrease in IFP at D1-TX and post-TX IFP after SBRT was <3% from the pre-TX. The mean post-TX IFP was ~1.5% higher than at D1-TX. The mean increase in IFV at D1-TX and post-TX from pre-TX was 48% and 21%, respectively, whereas post-TX decreased by 18% from the D1-TX.

K^trans^ values obtained from ETM and CFM estimated IFP and IFV values at three-timepoints (i.e., pre-TX, D1-TX, and D2-TX) are given in Table 1.

Pre-TX IFP and tumor volume (V_t_), which is calculated from the T_1_-weighted dynamic images, exhibited a strong positive significant correlation (ρ = 0.90, *p* = 0.005). At D1-TX, IFP and V_t_ showed a strong positive linear relationship (ρ = 0.71, *p* = 0.054), while at D2-TX, V_t_ and IFP were positively moderately correlated (ρ = 0.55, *p* = 0.13). CFM estimates of IFV, which is proportional to the gradient of p_i_ (Equation (4)), exhibited weak correlation with V_t_ at pre-TX (ρ = −0.38, *p* = 0.35) and moderate correlation at D2-TX (ρ = −0.63, *p* = 0.08), respectively. In contrast, IFV showed a strong negative correlation with V_t_ at D1-TX (ρ = −0.74, *p* = 0.046), indicating a slight change in IFP before the change in tumor volume.

**Figure 4:**
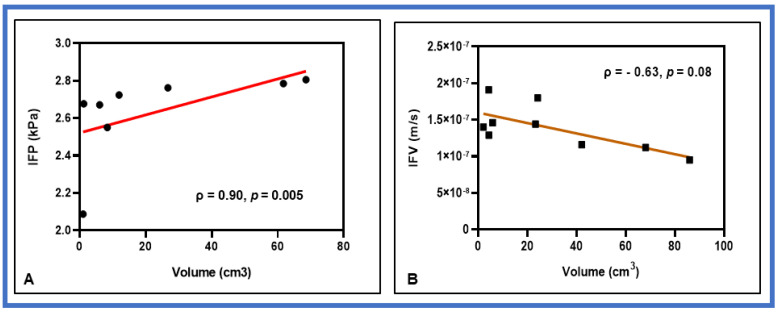
Representative correlation plots between CFM estimates of (**A**) interstitial fluid pressure (IFP) and tumor volume at pre-TX (**B**) interstitial fluid velocity (IFV) and tumor volume at D2-TX. IFP and IFV exhibited a strong positive and negative correlation with tumor volume at the respective time points. The solid red line represents the least square fit.

Figure 4 shows the representative correlation plots between the tumor volume V_t_ and CFM estimates of IFP and IFV.

**Figure 5:**
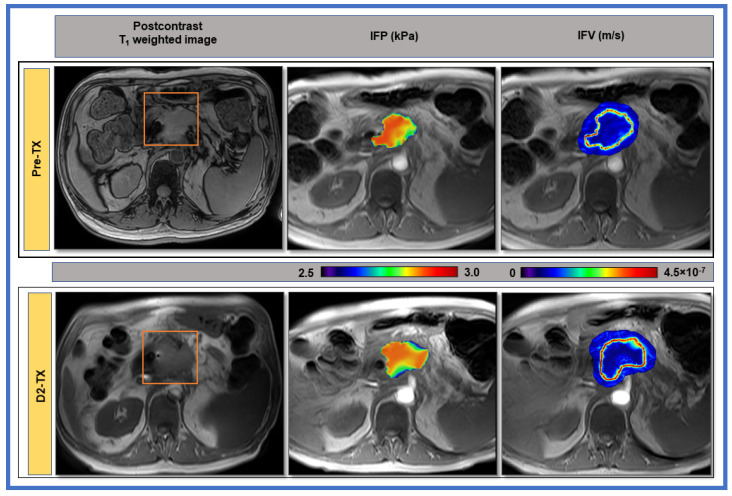
1st column: Representative pre- and post-stereotactic body radiation therapy (SBRT) T_1_-weighted post-contrast MR images of a patient (63 years, male) with PDAC shown in the body of the pancreas. The red rectangle delineates the region of interest. 2nd column: Computational fluid model estimates of interstitial fluid pressure (IFP). 3rd column: Interstitial fluid velocity (IFV) maps overlaid on pre-contrast T_1_-weighted images from PDAC patients at pre-and post-SBRT (D2-TX). A sharp drop in IFP at the tumor boundary results in evidently high IFV.

Figure 5 displays representative pre-and-post-SBRT (D2-TX) CFM estimates of IFP and IFV maps from a representative PDAC patient. Each of the IFP maps has physiologically reasonable values and shows regional heterogeneity. The IFP values fall off sharply at the tumor boundary as exudate fluid is absorbed by lymphatics in the surrounding tissue. In contrast, IFV (gradient of p_i_, Darcy Equation (4)) is outwardly directed from the tumor, achieving the highest velocities at the tumor boundary, where the change in IFP is, demonstrating that IFV within the tumor rim is not uniformly maximal.

## 4. Discussion

To the best of our knowledge, this is the first study assessing the predictive capabilities of CFM-simulated IFP and IFV using COMSOL in PDAC patients treated with SBRT. While modeling Equation (8), K^trans^ values estimated from ETM, describing the passive diffusion of CA into the EES within the voxel, were accounted for as an input for the influx of CA into the interstitium. In general, a model that reflects the underlying pathology is used through a model selection approach to describe the heterogeneous tumor tissue [19]. However, the accuracy and precision of the model-derived quantitative parameters depend on the spatial and temporal resolution, MR acquisition parameters (e.g., flip angle and repetition time, etc.), amount of CA injected, and the target tumor tissue under study. Well-established fluid-mechanical theory in porous media was applied to DCE data for noninvasive estimation of tumor IFP and exudate IFV at the tumor periphery. Rescaling of convective flux, Jv/V values, accounting for spatially varying capillary hydraulic conductivity, was applied, which is affected by the surface-to-volume ratio of the microvasculature, local tumor blood flow, vascular resistance, and IFP itself [43,52]. Accounting for potential spatially varying capillary hydraulic conductivity in the CFM approach would likely further improve IFP estimation, and consequently, IFV.

The CFM model assumes a poroelastic medium (ECM) that considers both fluid sources (vascular) and sinks (lymphatic drainage). The capillary hydraulic conductivity describes the convective flux of fluid across the capillary wall, which depends on the size of the capillary and pore size within the capillary. Provenzano et al. suggested that the increased pressure causes collapse of the vasculature and diminished diffusion into the tumor interstitium [26]. On the other hand, Chauhan et al. reported that IFP can only transiently exceed microvascular pressure but cannot compress tumor vessels [25]. Solid stress elevation in tumors due to high densities of cells and ECM can compress blood vessels. The reduction in mechanical forces is favorable for intratumoral diffusion and effective convective drug delivery. The K_H_ factor, relating the Darcy velocity and gradient of the IFP (∇p_i_), is affected by the porosity, viscosity of the interstitial fluid, the specific surface area of the porous media, and the geometrical structure of the ECM pores. Therefore, reliable estimation of K_H_ of the interstitium through which fluid travels is particularly vital in CFM simulation. Noninvasive CFM estimates of IFP need to correlate with the invasively measured IFP, which is not always readily available, to use CFM estimates of IFP and IFV as biomarkers in clinical practice. As a note, the simulation was performed using literature values for K_H_. Thus, K_H_ is not taking into account any effect of the treatment on the ECM composition and, hence, on its solid stress.

PDAC tumor vessels are moderately leaky [25]. One assumption is that SBRT treatment profoundly affects tumor vasculature, increasing vessel permeability and local tissue hydraulic conductivity. Park et al. reported that a single dose of 5–10 Gy to human tumor xenografts or rodent tumors causes relatively mild vascular damage. On the other hand, increasing the radiation dose to higher than 10 Gy/fraction induces severe vascular damage, resulting in reduced blood perfusion [63]. Cao et al., in a preclinical study, reported the stroma-directed therapy macromolecule PEGPH20 permeates across PDA vessels and acts on interstitial hyaluronic acid, HA. This consequently induces an increase in K^trans^, given that PDAC is poorly perfused before treatment, leading to relief of IFP and reopening otherwise collapsed microvasculature [23]. Thus, strategies aimed to mitigate mechanical forces might yield therapeutic benefits in PDAC. Bown et al. observed a 35% increase in ETM-derived K^trans^ value at 4 h after single radiation exposure in an orthotopic brain tumor model [64]. Herein, the ETM-derived mean ΔK^trans^_1-0_ value increased by 89% above the pre-TX level after initiation of SBRT. The mean ΔK^trans^_2-0_ values were increased 29% from the pre-TX, whereas ΔK^trans^_2-1_ values decreased by 31%, indicating mild and severe damage to microvasculature at D1-TX and D2-TX, respectively.

Tumor vasculature has abnormal organization, structure, and function characterized by heterogeneous blood flow and leaky vessels [65]. Vascular hyperpermeability and the lack of functional lymphatic vessels inside tumors elevate IFP in solid tumors, which can be inferred from the K^trans^ values, increasing perfusion with decreasing IFP (Table 1). A decreasing trend (<3%) of IFP in PDAC patients was observed for all time-points in PDAC patients treated with SBRT. In contrast, IFV increased by =<48% for all time-points. Improved fluid removal from the tumor EES would lead to a decrease in IFP. On the other hand, an increase in the tumor tissue K_H_ would increase the IFV through the tumor interstitium, which is assumed to depend on the structure and composition of the interstitial space [53]. Znati et al. concluded that reducing microvascular pressure is a plausible explanation for decreasing IFP after therapy [66]. The CFM estimated tumor IFP is uniform in the tumor interior and precipitously falls at the tumor boundary. In contrast, IFV becomes evident at the tumor boundary, which generally showed higher values in the tumor periphery than in central tumor regions. The IFP parametric maps exhibited intratumor heterogeneity in PDAC, even though the quantitative IFP values only changed relatively slightly (Figure 4).

The DCE-based CFM measurement exhibited an important relationship between IFP and tumor volume, despite the small sample size of PDAC patients. The lack of a significant and strong correlation across the time-points could be due to the limited number of tumors in the analysis. Previous studies have reported that the change in quantitative metric value, including IFP, could be observed prior to any macroscopic changes in tumor volume [66,67,68,69]. The present study also observed similar results, which could be due to microscopic alterations in the tumor microenvironment rather than the size of the tumor after SBRT. Prior clinical studies showed a similar trend between tumor IFP and volume [34,35]. Gutmann reported that IFP was 0.5–4.4 kPa for tumor volume ranging from 0.7 to 27 cm^3^ in 16 head and neck cancer (HN) patients [34]. Nathanson et al. also demonstrated that the IFP was 0.71–7.02 kPa for tumor diameters ranging 0.6–5.0 cm in breast ductal carcinomas. Yeo et al. reported a 68% decrease in median IFP values at mid-TX from the pre-TX in cervical cancer [37]. Chauhan et al. measured the IFP with WIN in the tumors of four treatment-naïve PDAC patients, and tumor IFP ranged from 0.81 to 2.23 kPa [25]. Our prior CFM modeling result showed the dependency of IFP on tumor volume in HN cancers [45], consistent with these studies [34,35]. In the preclinical study, Du fort et al. measured the IFP in mice using the WIN technique, ranging from 0.62 to 1.89 kPa, for tumor sizes between 5 and 9 mm in diameter [33]. Herein, it is important to note that a correlation between tumor size and IFP and IFV is tumor-specific [66].

Several studies have suggested that IFP may have important clinical implications for therapeutic advances in PDAC. The dense desmoplastic stroma of PDAC, containing a large amount of EMC that includes fibrillar collagen, fibronectin, laminin, and proteoglycans such as HA, may hinder the efficient delivery of chemotherapeutic agents [7,26]. Mechanisms postulated to underlie the relationship between IFP, and RT response includes the fact that high IFP leads to greater intratumoral hypoxia and may stimulate tumor angiogenic activity and vascular persistence, thereby contributing to radioresistance [70]. Tumor hypoxia directly impacts radiation response, since the presence of molecular oxygen is necessary to chemically fix DNA free radicals produced by ionizing radiation [71]. With regard to different fractionation schemes, tumor hypoxia likely has a greater impact on the treatment efficacy of SBRT where radiation is delivered in fewer fractions compared to conventionally fractionated radiation because reoxygenation of hypoxic tumor fraction is precluded by the relatively short treatment duration. On the other hand, SBRT using high fraction size has been suggested to damage tumor vasculature directly through its effect on endothelial cells [72]. Changes in the integrity of vasculature and mechanical forces due to SBRT are likely to be complex and directly impact IFP and IFV values and may correlate with long-term tumor response.

There are a few limitations in the present study. A larger patient cohort is warranted to validate these current findings. An unequal number of ROIs were used for the different time points because two patients did not participate in the longitudinal MRI exams. The noninvasively estimated IFP or IFV needs direct measurement with a standard invasive method to be validated as biomarkers in clinical practice. In future studies, the standardized DCE-MRI protocols need to include B_1_ inhomogeneity correction for analysis. Further work requires determining the spatially varying interstitial hydraulic conductivity and additional tissue-specific measurements, which can be challenging to procure through experimentation in clinical studies. Our noninvasive DCE-based computational framework offers significant scope for future expansion in radiation oncology and drug-therapy clinical trials.

## 5. Conclusions

The present study investigates imaging biomarkers, such as IFP and IFV, using a noninvasive DCE-based CFM approach. This method can provide important information regarding the tumor microenvironment for therapeutic advances in PDAC when standard invasive techniques are not readily available. After validation in larger patient cohorts, IFP/IFV estimates from CFM may help treating physicians to develop precision medicine in PDAC.

## Figures and Tables

**Table 1:** K^trans^, IFP, and IFV values at three-time points.

Parameter	Pre-TX	D1-TX	D2-TX	*p*-value
K^trans^ (min^−1^)	0.14 ± 0.06	0.27 ± 0.035	0.19 ± 0.06	*p* = 0.005 for Pre-TX vs. D1-TX, *p* = 0.01 for D2-TX vs. D1-TX, and *p* = 0.27 for Pre-TX vs. D2-TX
IFP (kPa)	2.63 ± 0.23	2.57 ± 0.19	2.60 ± 0.25	>0.05 (for all TX)
IFV (m/s)	(1.15 ± 0.50) ×10^−7^	(1.71 ± 0.75) ×10^−7^	(1.39 ± 0.31) ×10^−7^	*p* = 0.08 for Pre-TX vs. D1-TX *p* > 0.05 for all others TX

Note: IFP: Interstitial fluid pressure and IFV: Interstitial fluid velocity, Pre-TX: Pre-treatment, D1-TX: immediately after the first fraction of SBRT; D2-TX: Post-TX.

## Data Availability

The data presented in this study will be provided upon reasonable request.
